# Methicillin-Resistant *Staphylococcus Aureus* Septic Internal Jugular Thrombophlebitis: A Case Report

**DOI:** 10.5811/cpcem.42505

**Published:** 2026-03-07

**Authors:** Daniel Kowalczyk, George Ubiñas

**Affiliations:** University of Missouri, Department of Emergency Medicine, Columbia, Missouri

**Keywords:** septic thrombophlebitis, MRSA, case report

## Abstract

**Introduction:**

Lemierre syndrome is characterized by septic thrombophlebitis of the internal jugular vein, classically caused by *Fusobacterium necrophorum*. It is typically seen after an episode of pharyngitis where the palatine tonsils or peritonsillar mucosa is affected. It is thought to spread locally into the pharyngeal space toward the internal jugular vein.

**Case Report:**

A 42-year-old male with progressively worsening, atraumatic right-sided neck pain was discovered to have methicillin-resistant *Staphylococcus aureus* (MRSA) bacteremia, septic thrombophlebitis of the right dural venous sinuses, skull base osteomyelitis, and otomastoiditis.

**Conclusion:**

While septic thrombophlebitis of the dural venous sinuses and internal jugular vein is typically caused by *F necrophorum* and usually comes from local pharyngeal spread, community-acquired MRSA is an emerging cause of this pathology.

## INTRODUCTION

Lemierre syndrome, or septic thrombophlebitis of the internal jugular vein, is a rare diagnosis characterized by local infiltration of the lateral pharyngeal space by bacterial tonsillitis or pharyngitis. It was first described in 1936 by French physician André Lemierre. It is most commonly caused by *Fusobacterium necrophorum*, which can lead to significant Gram-negative bacteremia and sepsis. Even a viral pharyngeal infection can cause mucosal damage, allowing bacterial overgrowth and local spread. However, *F necrophorum* is only one of many bacterial species that cause internal jugular vein septic thrombophlebitis. Other described sources of local spread include parotitis, otitis, mastoiditis, or sinusitis. This case highlights the challenging nature of making this diagnosis along with broadened awareness of both atypical sources of this infection and atypical microorganisms causing infection, including many Gram-positive organisms.

## CASE REPORT

A 42-year-old male with no reported past medical history presented to the emergency department for evaluation of severe right neck pain. He reported a recent history of an ear infection on the right, treated with a course of antibiotics approximately one month prior. However, he reported persistence of right ear discomfort along with subjective fever and chills, severe nausea, subjective dysphagia and right-sided neck pain, posterior headache, and difficulty turning his neck side-to-side that had been worsening over the course of weeks. Upon arrival, the patient was afebrile at 36.6° Celsius, blood pressure144/80 millimeters of mercury (mmHg), heart rate 63 beats per minute, respiratory rate was 22 breaths per minute, and oxygen saturation was 98% on room air. His physical examination revealed three individual pustules seated on an erythematous base in the right ear with considerable erythema in the superior aspect of the right ear canal. His cranial nerve examination revealed a subjective decrease to light touch in the V1–V3 distribution of the right fifth cranial nerve.

A complete blood count (CBC), comprehensive metabolic panel (CMP), quantitative C-reactive protein (CRP) and erythrocyte sedimentation rate (ESR) were obtained. His white blood cell count was 13.6 x 10^9^/liters (L) (reference range: 3.5–10.5 x 10^9^/L), CRP was 3.65 milligrams per deciliter (mg/dL) (0–0.5 mg/dL,) and ESR was 63 millimeters per hour (mm/hr) (0–15 mm/hr).

After bloodwork had been performed, non-contrast computed tomography (CT) of the patient’s head along with CT angiography of the head and neck were performed, which demonstrated findings of right mastoid air cell opacification and vascular findings concerning for dural venous sinus thrombosis (Image A). Further CT and magnetic resonance imaging (MRI) later revealed skull base osteomyelitis, septic thrombophlebitis, and otomastoiditis (Images B and C).

Initially, after preliminary review of the labs and initial CT head and angiography of the head and neck, the decision was made to draw blood cultures and initiate treatment with ampicillin/sulbactam. The patient was admitted to the hospital. Multiple consulting services followed his case including otolaryngology, vascular neurology, neurosurgery, and infectious disease. Ampicillin/sulbactam was changed to vancomycin, meropenem and ciprofloxacin prior to magnetic resonance imaging (MRI). Upon completion of the MRI, the antibiotic regimen was changed to vancomycin, ceftriaxone, and metronidazole. He was started on a heparin infusion due to his thrombosis. The patient’s blood cultures returned positive for methicillin-resistant *Staphylococcus aureus* (MRSA). He had a peripherally inserted central catheter (PICC) placed and received vancomycin for a total of six weeks outpatient, and he was transitioned from heparin to apixaban.

One month after discharge from the hospital, he had a follow-up with infectious disease and neurosurgery. It was discovered on examination that he had a residual right cranial nerve XII deficit. He also was noted to have diminished hearing on the right as compared to the left. A repeat MRI showed improvement of the right-sided mastoiditis and resolved thrombosis in the right sigmoid sinus, right transverse sinus, and straight sinus. However, he did have findings of a chronically thrombosed right internal jugular vein and persistent demonstration of the aggressive osseous erosions and enhancement in the right occipital condyle. Overall, after completion of antibiotics, his clinical course improved.

*CPC-EM Capsule*

What do we already know about this clinical entity?

*Septic thrombophlebitis of the internal jugular vein (Lemierre syndrome) is usually secondary to oropharyngeal infection spreading to surrounding structures*.

What makes this presentation of disease reportable?

*This case was not secondary to the most common pathogen Fusobacterium necrophorum. It was caused by community-acquired methicillin-resistant Staphylococcus aureus*.

What is the major learning point?

*Community-acquired methicillin-resistant Staphylococcus aureus is becoming a well described pathogen in the literature as the culprit for Lemierre’s syndrome*.

How might this improve emergency medicine practice?

*Emergency physicians can consider adding vancomycin to the empiric regimen if this condition is discovered, especially in cases of suspected sepsis*.

## DISCUSSION

Lemierre syndrome, also known as septic thrombophlebitis of the internal jugular vein, is a rare complication of head and neck infection, most commonly oropharyngeal infection. The likely pathophysiology of how this syndrome arises is mucosal damage, allowing local bacterial invasion and seeding of the internal jugular vein, eventually leading to bacteremia.^1^ The most common cause of this infection is *F necrophorum;* however, other organisms including *Staphylococcus* are being described more often.^2^,^3^ While the typical source of this complication is oropharyngeal infection, we must consider other sources in the head and neck that are near the jugular venous system. The sources include parotitis, mastoiditis, sinusitis, orbital cellulitis, neck cellulitis, and otitis media as in our case.^2^–^5^

As described in a meta-analysis performed by Gore in 2020, MRSA was in the top five of the most commonly isolated organisms from blood cultures of patients with septic internal jugular thrombophlebitis. Their study also evaluated the overall mortality in patients who were anticoagulated or had vessel recanalization vs no anticoagulation. They found no statistically significant benefit to anticoagulation and that intravenous (IV) antibiotics were the mainstay of treatment tailored to blood cultures. Early IV antibiotic initiation is important in these cases as severe complications such as meningitis, septic pulmonary emboli, and septic cerebrovascular emboli have been described.^6^

Our patient was diagnosed with community acquired (CA)-MRSA bacteremia. Overall, the incidence of this pathogen is rising. Compared to its hospital-acquired counterpart (HA-MRSA), CA-MRSA has a different genotype and virulence profile.^7^ The HA-MRSA has a very limited number of IV antibiotic treatment options compared to CA-MRSA, which can be treated with oral options.^7^ Most commonly, CA-MRSA is a cause of skin and soft tissue infection; rarely can cause other infections including lymphadenitis, otitis media, otitis externa, mastoiditis, retropharyngeal abscess, preseptal cellulitis, and sinusitis.^8^ Risk factors for developing this infection include living in close group quarters, using illicit drugs, recent antibiotic administration, and immunosuppressive states such as HIV infection. 7 As in our case, delayed presentation and inadequate initial treatment of the inciting illness can lead to complex complications. It is important to maintain a heightened suspicion of alternative diagnoses when a patient presents with an otherwise benign illness that has not responded to conventional treatment. Finally, because of the increased incidence of CA-MRSA, if septic thrombophlebitis is discovered, we advocate for early initiation of broad-spectrum antibiotics to include MRSA coverage, not just *Fusobacterium*.

## Figures and Tables

**Image B:**
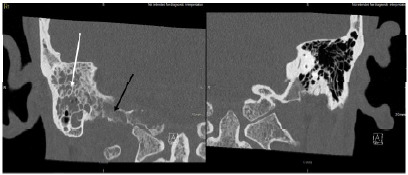
Coronal view computed tomography temporal/petrous bones re-demonstrating right mastoid air cell opacification (white arrow) along with aggressive osseous erosions of the occipital condyle and skull base (black arrow) with reference normal-appearing left-sided mastoid air cells, occipital condyle, and skull base.

**Image C:**
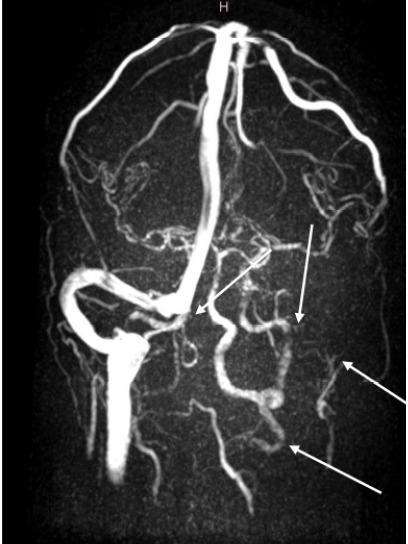
Magnetic resonance venogram brain in posterior-to-anterior orientation demonstrating complete opacification of the straight sinus, right transverse sinus, right sigmoid sinus, and right internal jugular vein (white arrows).
